# Smooth gap tuning strategy for cove-type graphene nanoribbons

**DOI:** 10.1039/d0ra02997a

**Published:** 2020-07-20

**Authors:** Tiago de Sousa Araújo Cassiano, Fábio Ferreira Monteiro, Leonardo Evaristo de Sousa, Geraldo Magela e Silva, Pedro Henrique de Oliveira Neto

**Affiliations:** Institute of Physics, University of Brasília Brazil pedrohenrique@unb.br; Theoretical and Structural Chemistry Group, State University of Goias Anapolis Goias Brazil

## Abstract

Graphene is a carbon-based material with an extensive range of promising properties. Since it does not present a bandgap, graphene is not suitable for optoelectronic applications. One possible way to open a gap is achieved by reducing graphene to its nanoribbon (GNR) form. Recently, a GNR with well defined cove-type periphery proper for large-scale production was synthesized showing an energy bandgap of 1.88 eV. In this work, we propose an edge termination strategy that allows for smoothly tuning the energy bandgap of cove-type GNRs by systematically changing the periodicity with which armchair-like and zigzag-like edges alternate. Using an extended two-dimensional Su–Schrieffer–Heeger tight-binding model we compare the effects of this edge termination process on lattice deformation with those arising from changes in nanoribbon width. Results show that modifications to the edges of cove-type GNRs are able to smoothly reduce energy bandgaps at the expense of losses in conjugation and increased morphological spreading. Energy band gap values starting from ≈3 eV to almost 0 eV were obtained. The flexibility provided by this gap tuning procedure places the cove-type GNR as an interesting candidate material for optoelectronic applications.

## Abstract 2

We investigated an edge transformation in cove-type graphene nanoribbons based on changing the balance of zig-zag and armchair chains.

## Graphical abstract

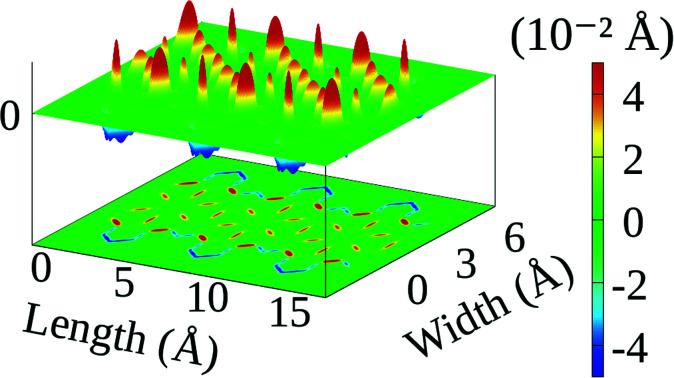

## I. Introduction

Graphene is a two-dimensional system composed of honeycomb lattices of carbon atoms. It hosts a broad set of interesting physical properties,^1–5^ resulting in the development of many graphene based applications.^6–9^ A drawback prevents the use of these materials in optoelectronic devices: the absence of an energy bandgap, which is the hallmark of semiconductor materials. However, bandgap opening can be achieved by means of several approaches, such as a doping procedures,^10–12^ which consist in the addition of non-carbon atoms into the lattice. The injection of these atoms induces a symmetry break in the system, leading to the appearance of a gap. Another way to engineer a gap opening is through the reduction in one of the dimensions of the graphene sheet until it reaches atomic scales (several angstroms). These quasi one-dimensional graphene strips are known as graphene nanorribons (GNRs).^13,14^ Due to their limited size, quantum confinement effects may arise, resulting in larger bandgaps. It is expected that GNRs will begin the next generation of semiconductor applications.^15–17^

The properties of GNRs are directly related to their geometries, with edge structure and width extension playing a key role on the electronic properties.^18^ Two edge shapes, known as zigzag (ZGNR) and armchair (AGNR) are specially relevant. These AGNRs are usually classified by the number *N*_a_ of atoms along their width (*N*_a_-AGNR) and may be divided in three families. These families are defined by *N*_a_ = 3*p* + 2, 3*p* + 1 and 3*p*, where *p* is a positive integer. Importantly, ZGNRs and AGNRs from the 3*p* + 2 family do not present appreciable bandgaps, but, on the other hand, AGNRs from 3*p* and 3*p* + 1 show semiconductor properties.^13^

**Fig. 1:**
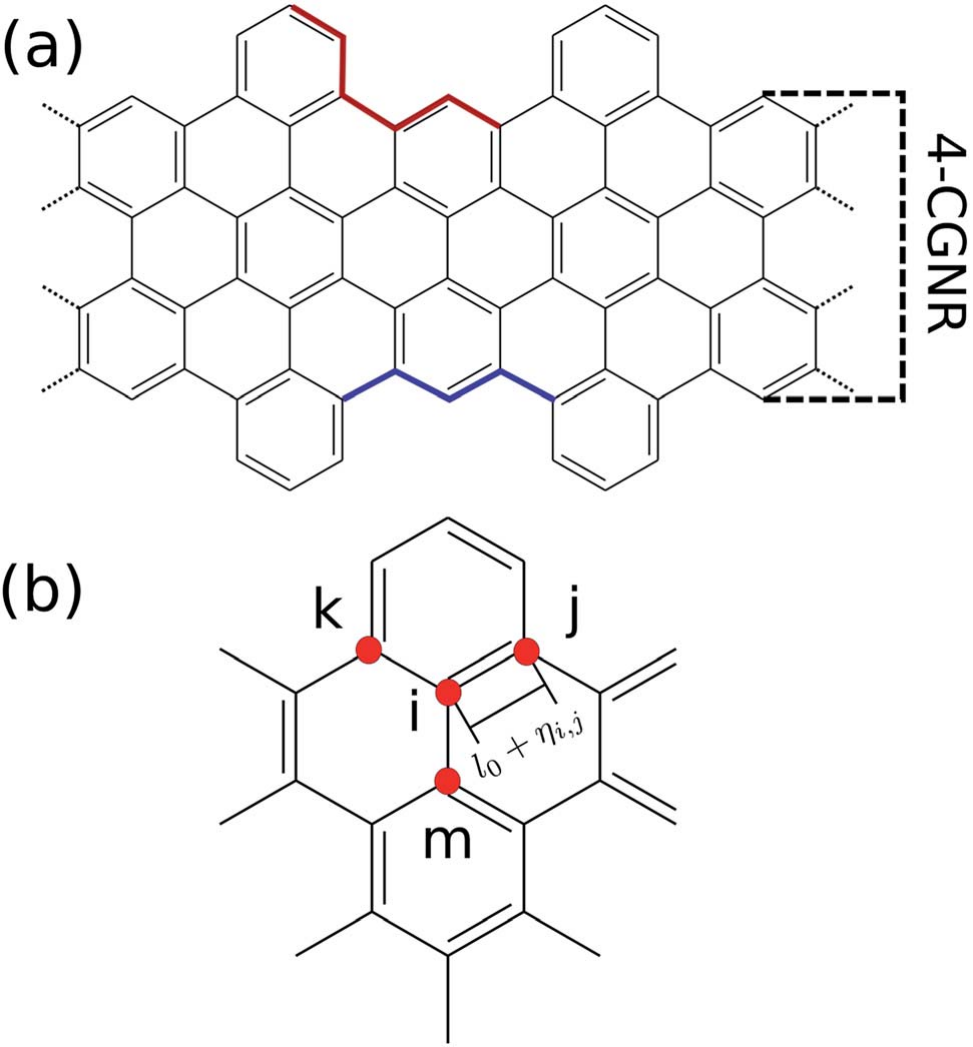
(a) Chemical structure of a 4 atoms wide cove-type GNR. The armchair-like and zigzag-like edges are highlighted in red and blue, respectively. (b) Site indexation used in the two-dimensional SSH model.

Recently, a graphene nanoribbon with a new edge termination was synthesized using a bottom-up liquid-phase procedure.^19^ The resultant GNR presented a cove-shaped edge (Fig. 1), which may be seen as a combination of the armchair and zigzag borders. Fig. 1(a) highlights examples of both border types inside the CGNR, with a sample of an armchair and zigzag border highlighted in red and blue, respectively. The synthesis technique employed relies on the use of smaller compounds through chemical reactions allowing an atomically precise design that mitigates structural defects and controls the proportion of each edge type. Known as cove-type GNR, or CGNR, this new nanoribbon architecture was reported as structurally well-defined and unusually long (>200 nm).^19^

Gap tuning can be performed by modifying the AGNR's width.^13,20,21^ However, since each AGNR family presents a particular gap dependence, this tuning procedure becomes complex. For instance, the difference between the energy bandgap of a 3-AGNR and 4-AGNR is about 0.18 eV.^13^ On the other hand, the gap variation between 4-AGNR and 5-AGNR is approximately 2 eV. As such, a smooth gap tuning procedure based on width changes in AGNRs is not possible.

Another possible strategy to tune energy gap relies on edge changes. The literature provides several successful attempts based on morphological transformation.^22–25^ Nanopores placed on GNR's lattice are an example.^26^ The defect produced by the hole induces the formation of V-shaped edges. This new border is a hybridization of armchair-like and zig-zag structures and controlling their relative amounts enables a smooth tuning procedure to be undertaken. As mentioned before, CGNRs can have its edge modified by a similar strategy. The question then arises as to how such changes may affect the electronic properties of these nanoribbons, as this could constitute a reliable method for gap tuning in GNRs.

To address the aforementioned issue, in this work, we simulated several cove-edge terminations to investigate both energy bandgap and conjugation changes in CGNRs. The nanoribbons were modelled using a two dimensional Su–Schrieffer–Heeger (SSH) model Hamiltonian. By means of a self consistent field approach, we evaluated the bond length distribution taking into consideration both electronic and phonon degrees of freedom. Our results show that changes in a single parameter that characterizes edge terminations leads to a monotonically decrease in the energy bandgap. By relating the bond length distribution pattern with the energy gap, this phenomenon is shown to be a consequence of the superposition of armchair and zigzag architectures. Gap values ranging from ≈3 eV to almost 0 eV were reached, showing that this method may be suitable for tailoring GNRs for very specific applications.

## II. Methods

The methodology applied in this work is similar to the methodology used in previous works of our group.^27–30^ The GNRs are simulated through the two dimensional extended SSH model, which starts with a Hamiltonian *H* = *H*_latt_ + *H*_tb_, where *H*_latt_ refers to the lattice Hamiltonian and *H*_tb_ corresponds to the electronic part. The lattice term reads1
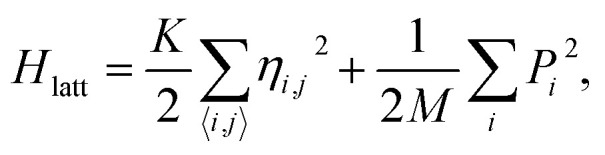
where *i* and *j* index neighboring sites, *K* is the harmonic oscillator constant, *P*_*i*_ is the momentum of *i*-th site, *M* is the site's mass and *η*_*i*,*j*_ is the relative displacement between the *i* and *j* neighboring sites (Fig. 1(b)). This means that, for instance, the distance between the site *i* and site *j* is *l*_0_ + *η*_*i*,*j*_, in which *l*_0_ is the bond length of a fully symmetric lattice (1.41 Å). The 〈 *i*,*j* 〉 term within the summation indicates a pair-wise sum.

As can be seen, the lattice part of the Hamiltonian corresponds to classical harmonic approximation. This is adequate since typical bond length variations in GNRs are usually not greater than 2%.^29^ On the other hand, the electronic terms are treated quantum mechanically by means of the second quantization formalism, modelling the π-electrons in a tight-binding approach. This electronic Hamiltonian is given by2
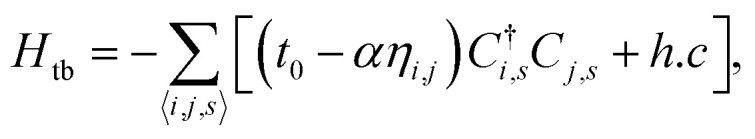
where the operator 
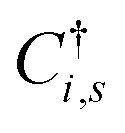
 is the creation operator of π-electron in *i*-th site with spin *s*. *C*_*i*,*s*_ is the corresponding annihilation operator. The lattice and electronic parts of the model are connected by the inclusion of an electron–phonon coupling (*α*) in the tight-binding's hopping term, in which *t*_0_ is the hopping integral for a symmetric lattice. The coupling between the electronic and lattice parts allows a more accurate description of these materials.

The description of our system is based on the set {*η*_*i*,*j*_} and the eigenvectors of *H*, {*ψ*_*k*_(*i*)}. However, these very set are required to evaluate the equations of motion. The problem can be solved by employing a self-consistent approach. An initial guess of {*η*_*i*,*j*_} (usually {*η*_*i*,*j*_} = 0) is chosen. Then, the Hamiltonian can be numerically calculated. With this procedure, the Schrödinger equation turns into an eigenvalue problem. The diagonalization process yields the eigenvectors {*ψ*_*k*_(*i*)}. The expected value of Lagrangian, 〈*L*〉 = 〈*Ψ*|*L*|*Ψ*〉, where |*Ψ*〉 is the Slater determinant is used in the Euler–Lagrange equation,3
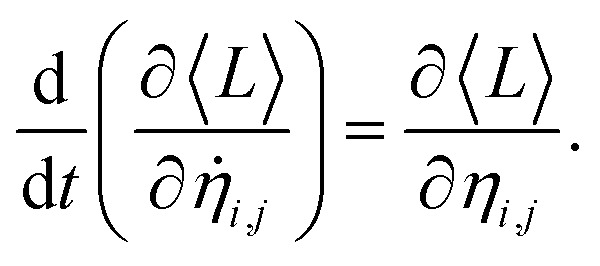


The solutions of these equations yield a new set of {*η*_*i*,*j*_}. The process is repeated. Once the iteration converges, the final set of {*η*_*i*,*j*_}, along with the associated eigenvectors and eigenvalues give the stationary state of the CGNR.

The Hamiltonian's parameters were chosen in accordance with previous works, for which *t*_0_ = 2.7 eV (ref. 31) and *K* = 21 eV Å^−2^.^32^ As for the electron–phonon constant, its evaluation is done through a semi-empirical procedure which will be described in the results session. Finally, as the number of carbon atoms on the width classifies armchair graphene nanoribbons, the same criterion will be applied throughout this work. For instance, the CGNR from Fig. 1(a) exhibits four carbons along the width axis, therefore, we refer to it as 4-CGNR. For the length direction, periodic boundary conditions are employed.

## III. Results

**Fig. 2:**
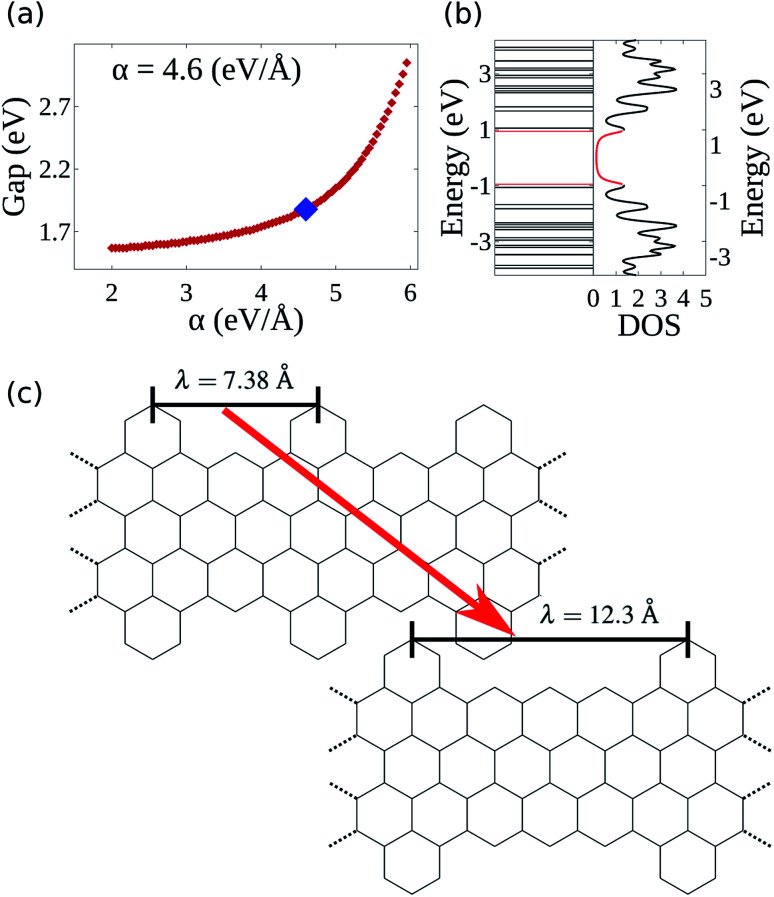
(a) Energy bandgap of the 4-CGNR of Fig. 1(a) as a function of the electron–phonon coupling constant (*α*). The coupling that coincides with the experimentally measured energy bandgap is the one which best represents the CGNRs. (b) Energy levels and corresponding density of states of a 4-CGNR with *α* = 4.6 eV Å^−1^. (c) Example of the edge termination process. *λ* indicates the periodicity of the alternation between armchair and zigizag-like edges.

As mentioned before, the electron–phonon constant *α* connects the dynamics of the π electrons with that of the lattice, affecting the energy bandgap. However, *α* cannot be easily measured directly,^28^ thus requiring an indirect method for its evaluation. Since the energy bandgap can be effectively determined experimentally, the appropriate electron–phonon constant is then determined to be the one that reproduces the experimental measure. For the cove-type 4-CGNR illustrated in Fig. 1(a), the optical bandgap is reported to be 1.88 eV.^19^ The gap is determined by the energy difference of eigenvalues of CGNR in a neutral state, which amounts to the optical gap. The formation of excitons is not considered as no relaxation process takes place. Fig. 2(a) shows the dependence between *α* and the energy bandgap, from which a coupling of 4.6 eV Å^−1^ is found to best reproduce the experimental results. AGNRs, for instance, present acceptable couplings values that range from 3.5 to 5.5 eV Å^−1^.^33^ Since the CGNR's *α* lies within this interval, the coupling between the lattice and electrons is observed to be roughly the same as in AGNRs.

The nanoribbon represented in Fig. 1(a) has its energy levels shown in Fig. 2(b). Where the valence and conduction bands consists of all lines below and above 0 eV, respectively. The highest occupied molecular orbital (HOMO) and the lowest unoccupied molecular orbital (LUMO) are highlighted in red, from which the 1.88 eV bandgap is obtained. It can be seen that some regions in the spectrum present an accumulation of energy levels in close proximity. This is better visualized by calculating the density of states as a function of energy (DOS). The DOS of the 4-CGNR of Fig. 1(a) is displayed on the right side of Fig. 2(b). Within the gap region, the DOS is identically zero. This forbidden zone arises as a result of the symmetry break induced by the edge termination of the cove-type GNR. The same effect occurs, for example, in polyacetylene, where the gap opens when a dimerized configuration is enforced. Furthermore, the appearance of an energy gap changes the profile of allowed states as well, with an increase in the number of energy levels near the HOMO and LUMO. This translates into the appearance of a peak close to those states, as seen in Fig. 2(b). Additionally the DOS tends to present high values even for energies away from the gap region. This behavior is shared with semiconductor AGNRs.^34^

**Fig. 3:**
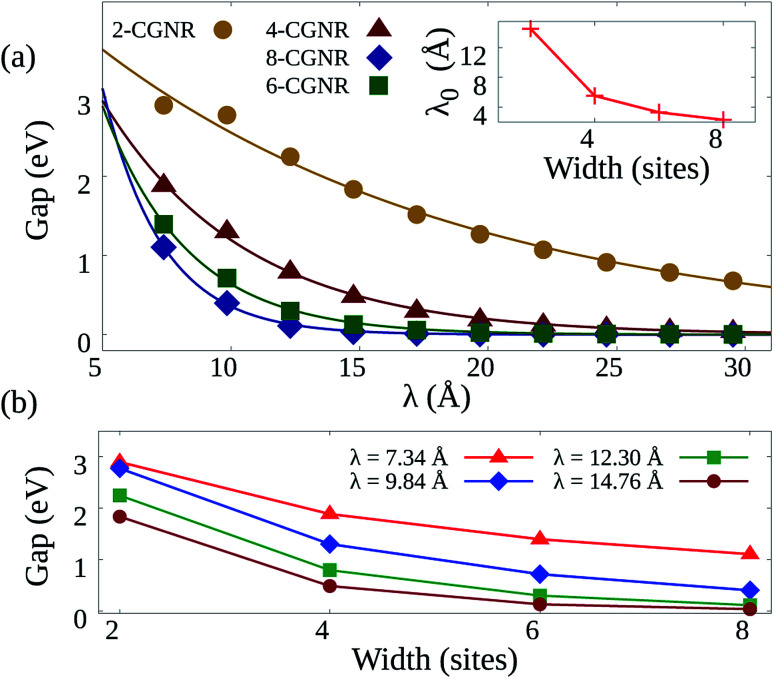
(a) Bandgaps as a function of *λ* for CGNRs of several widths. Each symbol represents a width and the lines are fits to the data considering an exponential decay. The inset highlights the behavior of the decay constant for each width. (b) Bandgap dependence on width for fixed *λ* values. Lines are guides for the eye.

Once the electron–phonon coupling has been determined, we may explore possible means of edge termination in CGNRs. As we have seen, the cove shaped edges are formed by alternating armchair and zigzag bond types. The periodicity of such alternation is measured by the parameter *λ*, which corresponds to the distance between two neighboring armchair-like edges (Fig. 2(c)). An increase in *λ* is equivalent to an increase in the proportion of zigzag to armchair edges. This zigzag bond injection procedure maintains the GNR with a cove-type periphery, but modifies its properties. Fig. 3(a) exhibits the effect of zigzag bond injection on the energy bandgap of CGNRs of different widths as a function of the parameter *λ*. As *λ* increases, the gap decays monotonically for all widths. This stems from the fact that as *λ* increases, the resultant nanoribbon resembles progressively more a pure zigzag GNR, which is known to present no energy bandgap. As such, the modification of the parameter *λ* in cove-type GNRs allows for a smooth and almost continuous gap tuning procedure.

The general decay profile of bandgaps with *λ* is shared by all widths. However, the decay rates are different for each CGNR, which results in different ranges of allowed gaps. For instance, 2-CGNR presents gaps ranging from about 0.68 eV to 2.90 eV. On the other hand, 4-CGNR starts at 1.88 eV and decays until nearly 0.04 eV. A steeper decay trend is seen for the broader nanoribbons, with 8-CGNR showing bandgaps that range from 1.10 eV until 0.00 eV. All of them converge close to 0 eV given a sufficiently long zig-zag edge chain. Pure ZGNRs present almost no gap when spin-polarization effects are not taken into account.^35–37^ Thus, the CGNR exhibits no significant additional contribution when it reaches the asymptotic region. This systematic decrease in gap magnitude is due to the reduction in quantum lateral confinement that takes place when nanoribbon width is increased.^38^ This effect can be quantified by fitting the data in Fig. 3(a) with a function *E*_gap_ = *E*_0_ exp(−*λ*/*λ*_0_), where *λ*_0_ is a characteristic length. This *λ*_0_ parameter is shown in the inset of Fig. 3(a) and decreases with nanoribbon width. Larger values of *λ*_0_ indicate the possibility of smoother tuning of the energy bandgap. As such, nanoribbon width may become a limiting factor for this tuning procedure.

Our model does not consider spin-polarized effects. This choice may have an direct influence on ZGNRs^13,35,36,39^ since spin-polarized simulations report this nanoribbon to be a semiconductor with magnetic properties.^37^ The width of each ZGNR inside the CGNR changes the gap value reached on the asymptotic regime, and it is reasonable to infer that the addition of spin-polarization will contribute to enlarger the final gap. Each CGNR converges to a specific gap value, depending on its width.

Density functional theory (DFT) studies regarding CGNRs are mostly concerned with geometries with *λ* = 7.38 Å.^40,41^ The gap values found in these works are in agreement with our results. For instance, 6-CGNR calculates a gap of 1.508 eV (ref. 42) while our simulated tight-binding model presented a gap of 1.394 eV. 8-CGNR's bandgap is estimated as 1.24 ± 0.03 eV.^43^ On the other hand, our calculations showed a gap of 1.104 eV. This accordance trend is shared with AGNR as well. For instance, LDA calculations show 5, 6, and 7-AGNR with, respectively, ≈0.5, 1.1, 1.65 eV.^13,21^ Our method evaluates the gap from the same geometries, respectively, 0.55, 1.75, 1.78 eV. This visible agreement shows the suitability of the presented methodology.

The effects of varying nanoribbon width for a given *λ* can be seen in Fig. 3(b). In wider nanoribbons, quantum confinement effects become weaker, reducing energy bandgaps accordingly. A similar dependence between gap and width size has been observed in AGNRs as well.^13^ However, it is also clear that changes in width do not produce bandgap reductions as smooth as those observed by increasing *λ*. This is so because, by preserving the edge structure, changes in width do not translate into a transformation from armchair-like to zigzag-like nanoribbons, which possess completely different bandgap properties. As such, controlling nanoribbon width does not constitute a tuning procedure as effective as controlling *λ*.

**Fig. 4:**
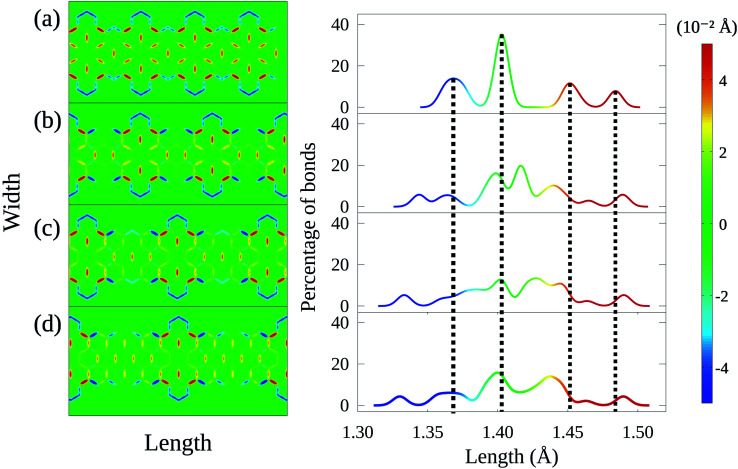
Bond length distortion heatmap and their corresponding histograms for 4-CGNR geometries with (a) *λ* = 7.38 Å, (b) *λ* = 9.84 Å, (c) *λ* = 12.3 Å, and (d) *λ* = 14.76 Å. Hot and cold colors indicate stretching and compression of bond lengths with respect to 1.41 Å, respectively. The histograms indicate that increases in *λ* induces changes in conjugation and increased morphological spreading.

Modifications in energy bandgap are associated with structural changes in graphene nanoribbons. In this sense, it is worth looking into how the edge termination described here affects the nanoribbon's morphology. Fig. 4 presents on the left heatmaps corresponding to bond length distortions for four 4-CGNRs of different *λ* (*λ* = 7.38, 9.84, 12.3 and 14.76 Å). Hot and cold colors indicate, respectively, stretching and contraction of bond lengths with respect to the 1.41 Å carbon–carbon bond length found in graphene. The magnitude of bond distortions reaches at most 0.04 Å, or roughly 3% of the original bond length, in agreement with similar works.^28,44^ On the right of Fig. 4, histograms present the distribution of bond lengths in each CGNR.

Results for the original 4-CGNR (Fig. 4(a)) are characterized by four well defined peaks in the histogram, which are marked by vertical dashed lines for the sake of comparison with the other cases. As it can be seen, this histogram indicates that almost 40% of the bonds in this nanoribbon do not suffer significant distortion. These bonds correspond mostly to the aromatic rings that lie at the center of the flower-like pattern seen in the heatmap on the left. These flower-like structures result from the stretching of the bonds between the aromatic rings and the exterior carbons. On the other hand, the edge bonds are seen to contract strongly, corresponding to the left-most peak observed in the histogram.

As *λ* increases, variation ensues. The well defined peak structure is seen to spread, with bond lengths being distributed more uniformly as *λ* grows larger (Fig. 4(b)–(d)). Although the behavior of edge bonds remains the same for all cases, the flower-like structures are no longer seen as the number of hexagonal rings diminishes. In fact, these structures are observed only in middle of regions whose edges are armchair-like. As such, we associate modifications in *λ* to changes in the conjugation of CGNRs.

**Fig. 5:**
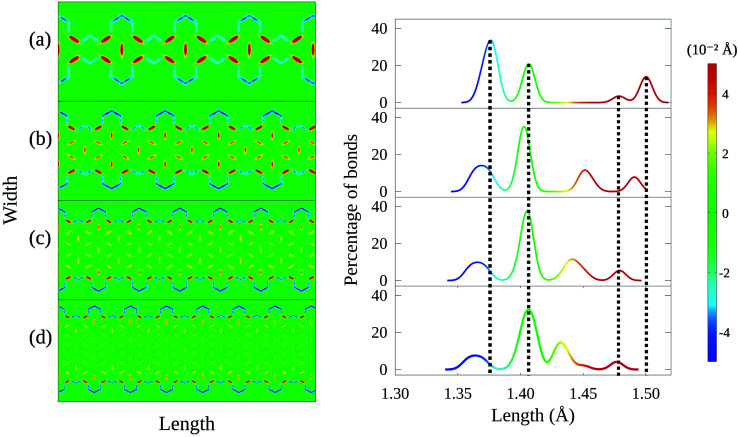
Bond length distortion heatmap and their corresponding histograms for (a) 2-CGNR, (b) 4-CGNR, (c) 6-CGNR and (d) 8-CGNR. All CGNRs shown here have *λ* = 7.38 Å. The histograms show that conjugation is preserved with width changes.

Finally, we perform a similar analysis considering increases in nanoribbon width for a fixed *λ*. Fig. 5 confirms the aforementioned relationship between conjugated bonds and the presence of armchair-like edges, as the flower-like structures are seen in all cases but the first, for which border effects are dominant. In addition, the histograms indicate that width increases may shift the position of the peaks, but do not produce the morphological spreading observed in the cases of varying *λ*. These results demonstrate that the edge termination process produces much more profound changes in the CGNR's properties, beyond bandgap modulation.

## IV. Conclusions

In conclusion, we have determined the electronic and morphological structures of cove-type GNRs by means of a two-dimensional extented SSH model. Comparing simulations with experimental results, it was possible to determine the electron–phonon coupling in this kind of nanoribbon to be 4.6 eV Å^−1^. An edge termination procedure was investigated and it was determined to constitute a smooth and nearly continuous gap tuning scheme as long as nanoribbon width is kept sufficiently small. Furthermore, it was demonstrated that this termination procedure leads to increases in morphological spreading accompanied by reduction in the conjugation of the nanoribbons. Finally, it was determined that even though width modifications do not result in morphological disorder, they are not as effective a tool for gap tuning.

Throughout this work, no heterogeneous cases were held. Thus, the real effects of the studied edge change are unknown to us. However, considering the results obtained, we may expect no substantial changes. The entire geometry will lose its symmetry but, locally, every combination of armchair–zigzag–armchair structures will remain itself as a cove-shaped edge. The tuning procedure will remain available. As for the lattice distortion profile, we would expect changes on the histogram peaks.

## Conflicts of interest

There are no conflicts to declare.

## Supplementary Material
